# Auto-phosphorylation Represses Protein Kinase R Activity

**DOI:** 10.1038/srep44340

**Published:** 2017-03-10

**Authors:** Die Wang, Nicole A. de Weerd, Belinda Willard, Galina Polekhina, Bryan R. G. Williams, Anthony J. Sadler

**Affiliations:** 1 Centre for Cancer Research, Hudson Institute of Medical Research, Clayton, Victoria, 3168, Australia; 2 Centre for Innate Immunity and Infectious Diseases, Hudson Institute of Medical Research, Clayton, Victoria, 3168, Australia; 3 Department of Molecular and Translational Science, Monash University, Clayton, Victoria, 3168, Australia; 4 Proteomics and Metabolomics Laboratory, Lerner Research Institute, Cleveland Clinic, Cleveland, Ohio, 44195, USA

## Abstract

The central role of protein kinases in controlling disease processes has spurred efforts to develop pharmaceutical regulators of their activity. A rational strategy to achieve this end is to determine intrinsic auto-regulatory processes, then selectively target these different states of kinases to repress their activation. Here we investigate auto-regulation of the innate immune effector protein kinase R, which phosphorylates the eukaryotic initiation factor 2α to inhibit global protein translation. We demonstrate that protein kinase R activity is controlled by auto-inhibition via an intra-molecular interaction. Part of this mechanism of control had previously been reported, but was then controverted. We account for the discrepancy and extend our understanding of the auto-inhibitory mechanism by identifying that auto-inhibition is paradoxically instigated by incipient auto-phosphorylation. Phosphor-residues at the amino-terminus instigate an intra-molecular interaction that enlists both of the N-terminal RNA-binding motifs of the protein with separate surfaces of the C-terminal kinase domain, to co-operatively inhibit kinase activation. These findings identify an innovative mechanism to control kinase activity, providing insight for strategies to better regulate kinase activity.

Control of enzyme activity by phosphorylation of protein hydroxyls is universal in biology. The collective kinome in eukaryotes regulates enzyme activity by phosphorylating protein substrates at tyrosine, serine or threonine residues. This phosphorylation alters the electrostatic properties of an enzyme to modify its conformation to activate or inhibit the enzyme, induce interactions with other proteins, and to stabilize or, alternatively, promote degradation. All cellular processes, including cell cycle, growth, metabolism, gene expression, cell differentiation and death, as well as responses to the environment, are orchestrated by protein kinases. This utility means that there is intense interest in developing pharmaceuticals to regulate the activity of kinases. This has impelled efforts to determine the structure and function of kinases and their distinct attributes that might enable specific therapy.

Structural analysis of many different protein kinase domains has identified that they adopt a broadly similar conformation when active. In contrast to this equivalence, different kinase domains appear to adopt diverse conformations in their inactive state^1^,^2^. Fitting with this, diverse mechanisms of auto-inhibition have been identified for different kinases^3^,^4^. Deciphering these different inactive states and understanding the mechanisms that maintain them is crucial to the design of inhibitors that might enable more specific therapeutic intervention to counter pathology caused by aberrant kinase activity. However, determining the state of inactive kinases and the mechanisms that impose auto-inhibition is challenging because of the dynamic movements that are inherent in these systems. We conducted a series of detailed biochemical experiments that probed different states of the protein kinase R (PKR) to determine the mechanisms that regulate its activation.

PKR is a member of a small family of kinases that induce a universal translational checkpoint, which enables eukaryotic cells to respond to environmental stress. Each member of this kinase family responds to separate stress stimuli to phosphorylate the eukaryotic translation initiation factor 2α (eIF2α), thereby inhibiting recharging of the initiation complex and suppressing general protein translation. PKR responds to duplex RNA molecules that are characteristic of viral transcripts. Accordingly, PKR constitutes an important arm of the antiviral response, which is underscored by its induction by the type I and III interferons. PKR is otherwise constitutively expressed^5^. This mandates that the kinase be tightly controlled at homeostasis to permit normal gene expression.

PKR encodes an amino-terminal RNA-binding domain, which consists of tandem RNA-binding motifs (RBM1 and RBM2), and a carboxyl-terminal kinase domain that are linked by unstructured regions. It was initially asserted that the activity of PKR was regulated by an auto-inhibitory interaction between RBM2 and the kinase domain^6^,^7^,^8^. The precise mechanism of how this interaction repressed the kinase was not determined. However, it was proposed that the suppressive intra-molecular association was relieved through binding to duplex RNA, initially by the unrestrained RBM1, then with co-operative binding pulling the RBM2 free from the kinase domain. This is followed by concomitant dimerization and auto-phosphorylation, with requisite phosphorylation of threonine residues within the catalytic loop^9^. However, more recent studies refute the auto-inhibitory mechanism, instead asserting that PKR exists as a monomer in an open conformation without activity until protomers co-localize on RNA via binding at their amino-terminus, which then enables trans-phosphorylation and subsequent formation of the active dimeric enzyme^10^,^11^,^12^,^13^.

Here we identify novel processes in the regulation of kinase activity. Our findings corroborate that PKR is controlled by auto-inhibition. However, in contrast to the initial report of the mechanism, we demonstrate that both motifs in the RNA-binding domain are involved. Unexpectedly, we identify that auto-phosphorylation of RBM1 is required to instigate the intra-molecular interactions with the kinase domain. This interaction alters the ternary state and activity of the protein. Our findings identify another innovative mechanism that has evolved to control the activity of kinases and provide insight for a strategy to develop inhibitors against kinase activity.

## Results

### Phosphor-residues in RBM1 repress PKR activity

**Figure 1:**
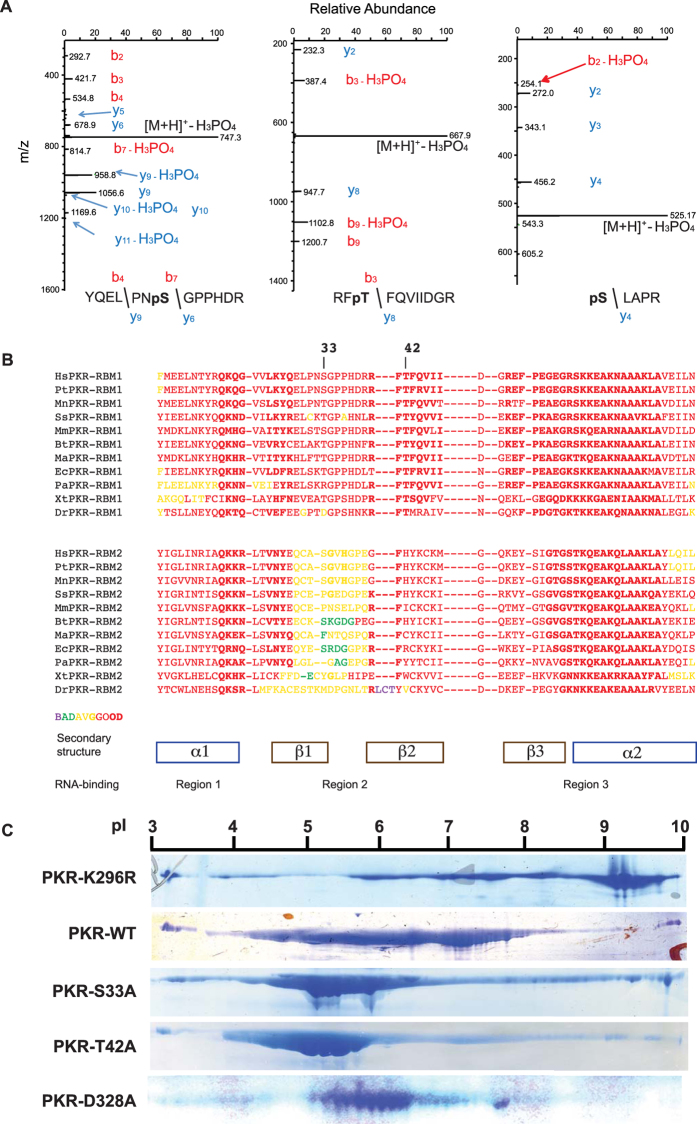
Phosphor-residues in RBM1 repress auto-phosphorylation of PKR. (**A**) LC-MS/MS analysis of the tryptic digest of PKR identifying phosphor-S33 and -T42 residues as doubly and phosphor-S242 as singly charged peptides, with m/z ratios of 795, 717 and 623 Da, respectively. The fragmentation spectra of the peptides are dominated by loss of H_3_PO_4_ from the precursor ion, which is indicative of the presence of a pS or pT residue and the mass of this ion is consistent with the YQELPNSGPPHDR, RF**T**FQVIIDGR or SLAPR + PO_3_ peptides. The site of modification is evident by the mass of the: N-terminal b_4_ and b_7_ ions, which is consistent with modification on S33 (YQELPNpSGPPHDR); N-terminal b_3_ ion, which is consistent with modification on T42 (RFpTFQVIIDGR); and C-terminal y_4_ ion, which is consistent with modification on S242 (pSLAPR), as indicated. (**B**) An alignment of the amino acid sequences of the PKR RBM1 (above) and RBM2 (below) from the indicated species; Hs = *Homo sapiens*, Pt = *Pan troglodytes*, Mn = *Macaca nemestrina*, Ss = *Sus scrofa*, Mm = *Mus musculus*, Bt = *Bos taurus*, Ma = *Mesocricetus auratus*, Ec = *Equus caballus*, Pa = *Pteropus alecto*, Xt = *Xenopus tropicalis*, Dr = *Danio rerio*. A more extensive alignment to RBMs from proteins other than PKR is shown in Supplementary Figure S1. (**C**) The indicated recombinant PKR proteins electrophoretically separated by their isoelectric point (pI), then visualized with the Coomassie brilliant blue stain.

Activation of PKR is accompanied by and contingent upon auto-phosphorylation. To gain insight into this process, we conducted mass spectroscopic analysis to identify phosphor-residues in recombinant human PKR produced in *Escherichia coli* and purified as described by us previously^14^. Mass spectrometry was conducted directly on the purified protein or after enrichment of phosphorylated peptides by metal affinity chromatography. Detection of a phosphate by direct analysis requires that a high quotient of molecules have the specific modification, and so, this approach distinguishes major phosphorylated sites. Although numerous phosphorylation sites were detected in enriched peptides, only three phosphor-residues were detected in the direct analysis. The residues identified were a serine and a threonine residue at positions 33 and 42, respectively, at the amino-terminus and a serine residue 242 within the linker region between the RNA-binding and kinase domains (Fig. 1A). The S33 and S242 residues had previously been identified as phosphorylation sites^15^,^16^,^17^. Comparison of the amino-terminal RNA-binding region of PKR from diverse species shows that the serine and threonine residues are conserved in the first RBM1 and not in the second RBM2 of PKR from diverse species, or among RBMs from other proteins (Fig. 1B and Supplementary Figure S1).

To determine the consequence of the phosphorylation of S33 and T42, these residues were mutated to alanines and the degree of auto-phosphorylation assessed as a measure of kinase activity. Auto-phosphorylation was assessed as the charge of the recombinant protein by isoelectric focusing. An unphosphorylated kinase-dead PKR molecule, in which the lysine residue number 296 that catalyzes transfer of the phosphate from ATP to a substrate is mutated to an arginine (K296R)^18^, focuses as a peptide above pH 9 (Fig. 1C). In contrast, the wild-type PKR focuses between pH 4 and 8 (Fig. 1C). This range in pH is consistent with the wild-type protein being variably auto-phosphorylated^7^,^19^. Surprisingly, the S33A and T42A mutant peptides carry an even stronger charge, focusing at between pH 4 and 6, consistent with these proteins being more highly phosphorylated (Fig. 1C). As a comparison, we evaluated the consequence of mutating the asparagine at position 328 (D328A), which has been reported to produce a constitutively active PKR^20^. This shows that substitution of an alanine at positions 33 or 42 within the RNA-binding domain has a more appreciable effect on PKR auto-phosphorylation than the previously reported activating mutation within the kinase domain.

As kinase activity was advanced by mutation of the S33 and T42 to a non-phosphorylatable residue, it suggests that, paradoxically, auto-phosphorylation at these sites represses activity. As auto-phosphorylation is crucial in establishing the conformation of the active enzyme, we conducted a series of biochemical experiments to assess whether modifying S33 and T42 within RBM1 affects the ternary structure of PKR.

### S33 and T42 phosphor-residues affect the exposure of the RNA-binding domain

**Figure 2:**
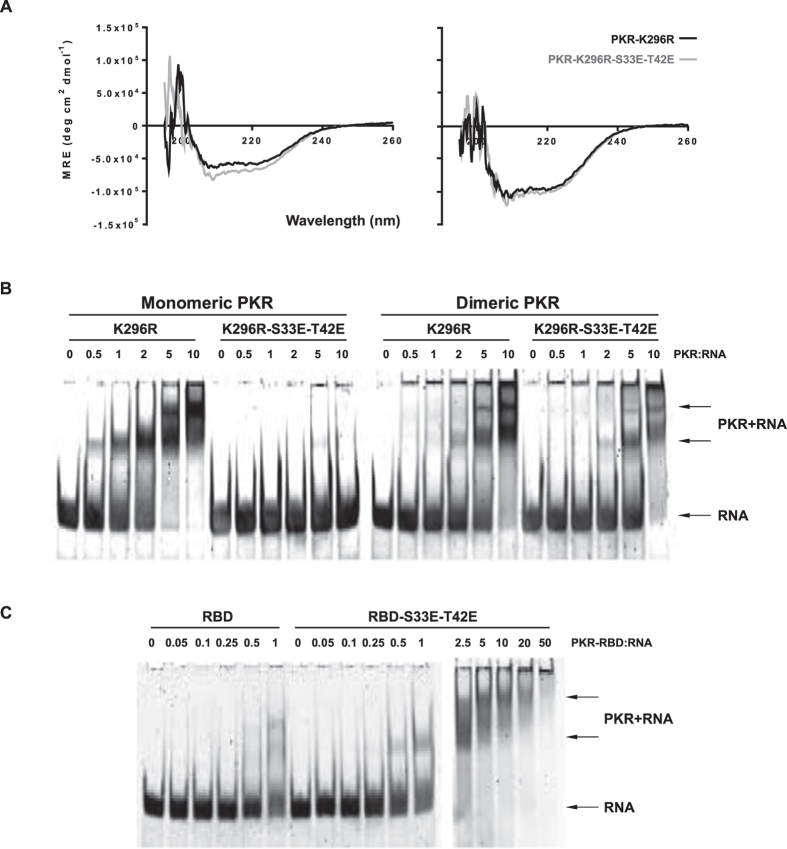
Phosphor-S33 and -T42 affect the exposure of the RNA-binding domain of PKR. (**A**) Comparisons of the secondary structure of the indicated recombinant PKR protein constructs, purified as either a monomer (on left) or dimer (on right) by circular dichroism. (**B**,**C**) Free HIV-1 TAR-RNA (RNA) and PKR-bound RNA (PKR+RNA) separated by electrophoresis then visualized with SYBR Gold nucleic acid stain. Increasing concentrations at the designated molar ratios of the indicated PKR constructs purified as either a monomer (gel on left) or dimeric full-length molecule (on right of B), and the truncated RNA-binding domain (RBD) of PKR (**C**). An extended titration of the S33E-T42E mutant RBD (2.5 to 50 times that of the TAR RNA) is included to demonstrate the complete capture of free RNA (on right of C).

Since RNA-binding at the N-terminus activates PKR, we examined a role for S33 and T42 in this function. The RBM binds RNA at three regions: (1) through a conserved glutamate from the helix-α1; (2) residues in the motif GPxH from the β1-β2 loop; and (3) positively charged residues in the motif KKxAK at the N-terminus of the helix-α2 (Fig. 1B). As the S33 and T42 do not constitute these residues, they would not be predicted to alter RNA binding. In keeping with this prediction, it was previously reported that mutating the S33 residue did not alter RNA-binding^16^. To assess the consequence of the specific phosphorylation of S33 and T42 from other phosphor-residues, we generated a recombinant protein that replaced S33 and T42 with phosphor-mimetic glutamic acids within the kinase-dead (K296R) molecule. As we have previously reported for the wild-type PKR^21^, this mutated protein was purified by liquid chromatography as both a monomer and a dimer. We compared the overall fold of the single (K296R) and triple (K296R-S33E-T42E) mutant proteins by circular dichroism. Both proteins show minima at 208 and ~220 nm, a characteristic of proteins with an alpha-helical structure, as has been established for PKR. Since the far-UV spectral scans for both proteins show comparable mean residues ellipticities across the wavelength range measured (190 to 260 nm), our data suggest that these proteins have equivalent secondary structures (Fig. 2A). However, in contrast to the single mutant (K296R), the monomeric triple mutant PKR (K296R-S33E-T42E) demonstrated a marked reduction in RNA-binding (Fig. 2B). Strikingly, RNA-binding was restored in the triple mutant protein that was isolated as a dimer (Fig. 2B). Therefore, the disruption of RNA-binding is not due to the mutations *per se*. This was confirmed by RNA-binding assays with truncated peptides that encompass just the RNA-binding domain (Fig. 2C). As the obstruction to RNA-binding in the full-length monomeric molecule is relieved by dimerization, it suggests an association between the RNA-binding domain and the dimer interface at the N-lobe of the kinase domain^22^. This intra-molecular association is relieved, freeing the RNA-binding domain, in the dimeric molecule through the alternative engagement of the N-lobe in the inter-molecular association between protomers. Accordingly, we propose that introducing a charge at the S33 and T42 residues induces an intra-molecular interaction between RBM1 and the N-lobe of the kinase domain.

### S33 and T42 phosphor-residues affect the state of the linker region of PKR

**Figure 3:**
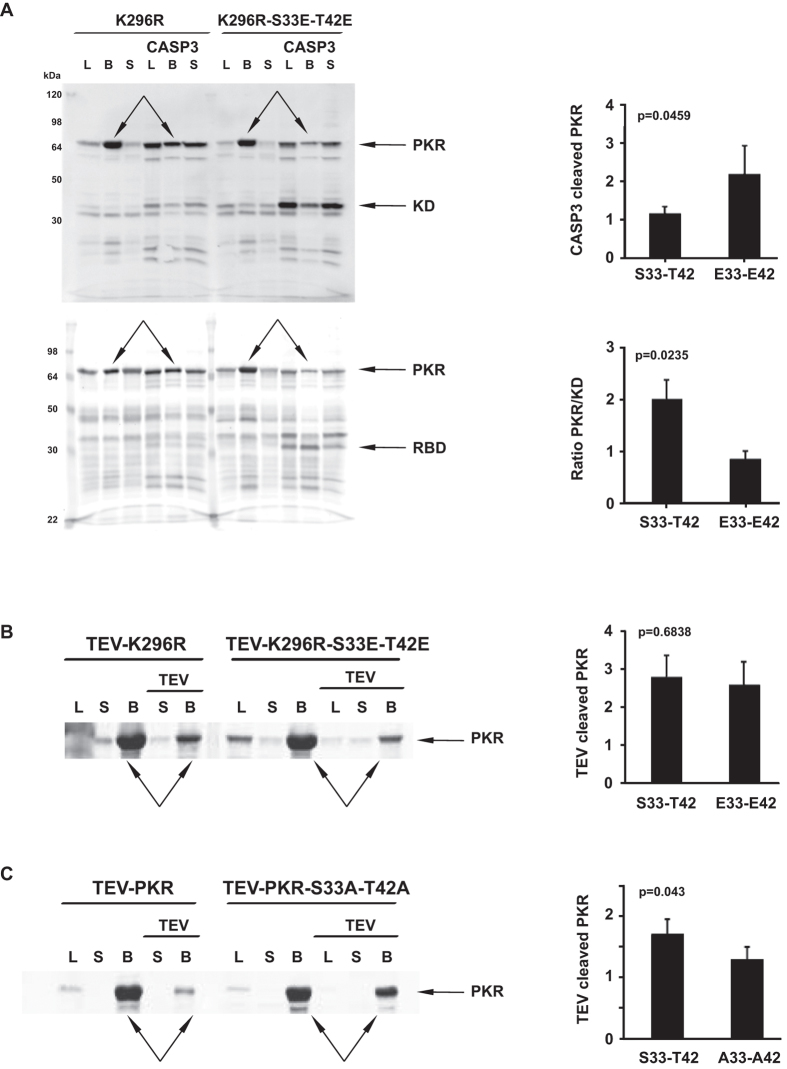
Phosphor-S33 and -T42 affect the state of the linker region of PKR. (**A** to **C**) Visualization of proteolytic digestion of the indicated PKR constructs with caspase-3 (**A**) or TEV (**B**,**C**) by immunoblot detection of the full-length or truncated kinase domain (KD) or, alternatively, RNA-binding domain (RBD) with anti-PKR antibodies specific for each domain of PKR. The immunoblot probes the whole bacterial lysate (L) or proteins captured on anti-His beads (B) or that remain in the capture-bead supernatant (S). The extent of proteolytic cleavage from repeated experiments is quantitated in the graphs on the right, as the amount of cleavage of the full-length PKR (CASP3 cleaved PKR (n = 3) or TEV-K296R (n = 3) or TEV-PKR (n = 7) cleaved PKR), with the protein bands measured indicated with arrows on the western blot or, alternatively, as the ratio of the full-length protein to the truncated product (Ratio PKR/KD (n = 6)). Student’s *t* test was used to calculate the *p* values from the data produced with the indicated PKR constructs.

A putative intra-molecular association would be expected to alter the state of other regions of PKR outside of the RNA-binding domain. The linker region between the RNA-binding and kinase domains contains a caspase-3 cleavage site at asparagine residue number 251^23^. We reasoned that an intra-molecular association might be discerned through altered proteolysis at this site. This analysis shows that the triple mutant (K296R-S33E-T42E) protein was more susceptible to caspase-3 cleavage than the single mutant (K296R) PKR (Fig. 3A and Supplementary Figure S2).

The experiment was repeated to confirm the specificity of the cleavage site by engineering a unique protease site within the linker. A tobacco etch virus (TEV) protease cleavage site was introduced at residue number 225 by mutating the amino acid sequence DSLNSSS to ESLYFQS. The influence of mutating S33 and T42 to glutamic acids for proteolysis was reduced in this TEV-PKR molecule (Fig. 3B). Although we did not quantify this effect, the TEV-PKR appeared to be more active than the wild-type kinase. This is in keeping with reports of an involvement of the linker in regulating PKR activity^24^. We also tested the consequence of the opposing mutation, to introduce unphosphorylatable alanine residues into the TEV-PKR. Mirroring the effect of the glutamic acid mutant on caspase-3 cleavage, the alanine mutant was less susceptible to proteolysis (Fig. 3C).

Accordingly, the phosphor-S33 and -T42 residues change the state of the linker region that links the RNA-binding and kinase domains.

### S33 and T42 phosphor-residues affect the state of the kinase domain

**Figure 4:**
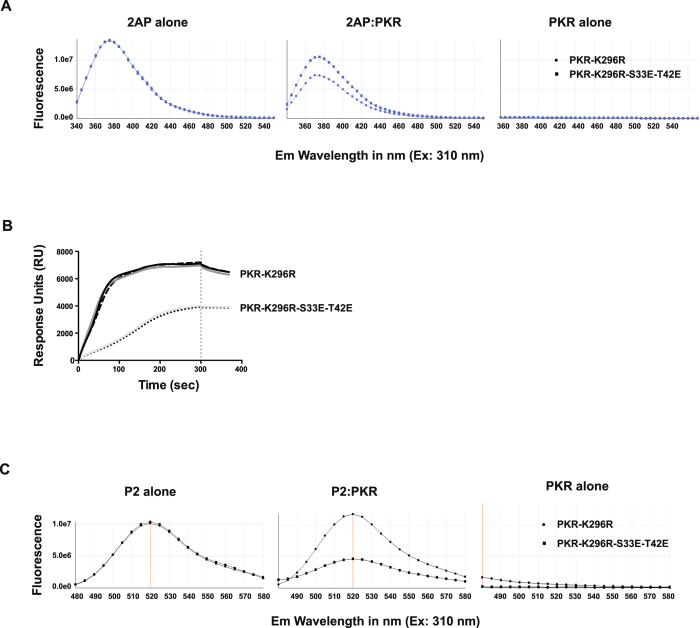
Phosphor-S33 and -T42 affect the state of the PKR kinase domain. (**A**) Measures of the comparative *in vitro* binding of 2-aminopurine nitrate (2AP) to the indicated recombinant PKR protein by assessing changed fluorescence from the molecule. The graphs show that the separate PKR constructs differently diminished 2AP-fluorescence at a ratio of 1:2 of 2AP to PKR. The consequence of a broader range of concentrations of PKR and of alternative PKR constructs are shown in Supplementary Figure S2. (**B**) Absorption resonance curves for ligand coupling of the indicated recombinant PKR proteins, demonstrating that the phosphor-residues generate distinct profiles. SPR experiments were carried out on a ProteOn XPR36 (Bio-rad Labs) using an HTE chip for His-tagged proteins and TBS as the running buffer. PKR proteins were immobilized to the nickel-activated chip via their His-tags after dilution to 50 μg/ml. Data shown are technical replicates from representative duplicate experiments and show the Response Units (RU) measured from the surface of the chip over the course of the sunitinib coupling (0 to 300 sec) to the washing step (300 sec and beyond), indicated by the vertical dashed line on the graph. Surface plasmon resonance (SPR) sensorgrams of the experiment between the PKR and sunitinib are shown in Supplementary Figure S3. (**C**) Measures of the comparative binding of a FITC-tagged peptide (P2) to the indicated PKR constructs by assessing fluorescence. The consequence of a broader range of concentrations of PKR and of alternative PKR constructs for FITC-P2 fluorescence is shown in Supplementary Figure S4.

To detect whether the effects of S33 and T42 residues extend to the kinase domain, we measured its association with the nucleoside analogue 2-aminopurine^25^,^26^. This nucleoside analogue inhibits the activity of PKR by competing for ATP binding within the catalytic pocket of the kinase domain^27^. The relative binding of a 2-aminopurine nitrate salt was assessed by measuring the degree to which the fluorescence of this free ligand was quenched by its association with PKR. The levels of fluorescence suggest that mutating the S33 and T42 residues to glutamic acids decreased the extent of 2-aminopurine binding (Fig. 4A and Supplementary Figure S2). Accordingly, phosphor-residues in the RBM1 alter the state of, or access to, the ATP-binding pocket of the kinase domain.

We conducted a second ligand binding experiment that measured the extent of binding of the non-ATP competitive kinase inhibitor sunitinib, which has previously been reported to bind to PKR^28^. The extent of this interaction was assessed by surface plasmon resonance spectroscopy. The sensorgrams of the association between the ligand (PKR) and analyte (sunitinib) were not able to resolve this association (Supplementary Figure S3). However, the absorption resonance curves for coupling of the single (K296R) and triple (K296R-S33E-T42E) mutant proteins differed, which is consistent with differences in their ternary structure (Fig. 4B).

Next, we tested an association between PKR and a peptide inhibitor, coded P2, which was reported to bind to the kinase domain outside of the catalytic pocket^29^. To enable quantification of its association with PKR, we linked fluorescein isothiocyanate (FITC) to P2 via an amino acid linker. As it was detached from the peptide by the linker, it was envisaged that the fluorophore would not be directly bound in the interaction, and so, we did not expect fluorescence quenching. In point of fact, fluorescence was increased in the association with the single mutant (K296R), presumably due to resonance energy transfer from aromatic residues within PKR (Fig. 4C). Contrasting this, fluorescence was decreased in the interaction with the triple mutant (K296R-S33E-T42E). This effect was strongly influenced by the ratio of the peptides (Supplementary Figure S4). Accordingly, the residues at positions 33 and 42 within the RBM1 markedly altered the state of the kinase domain or directly competed to alter the association with P2.

Together these data demonstrate that the phosphor-S33 and -T42 residues in RBM1 have allosteric effects that change the state of the following linker region and kinase domain. Together with the preceding findings, this is consistent with a putative intra-molecular interaction.

### Modelling the interaction between RBM1 and the kinase domain

**Figure 5:**
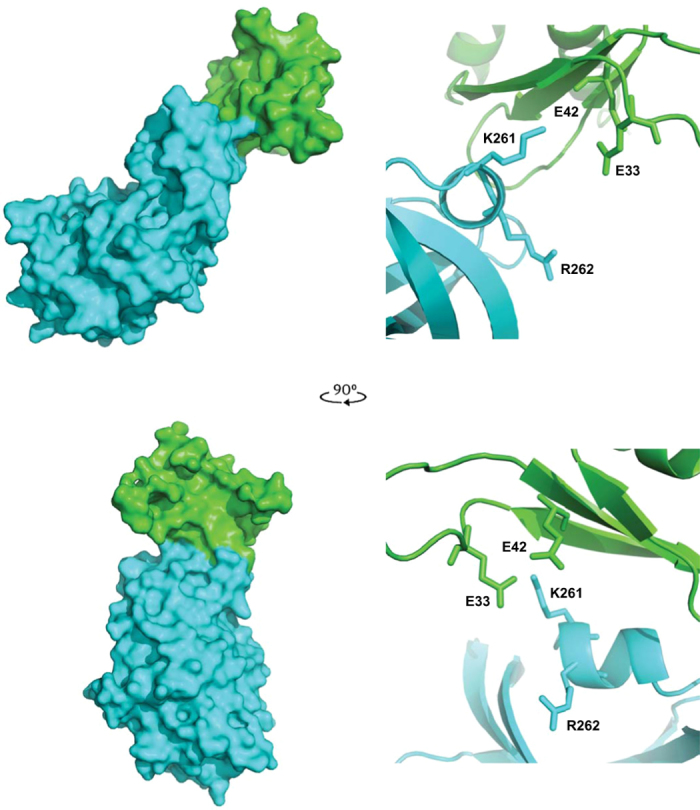
Modelling the interaction between RBM1 and the kinase domain. The predicted association between the PKR kinase domain (cerulean) in complex with the RBM1 (green) shown as a space-filling model (on left). Two perspectives of the complex are shown at right angles to each other. The RBM1 is predicted to associate with the N-lobe of the kinase domain, which faces the viewer in the lower perspective. Details of the docking orientation of the RBM1 with the kinase domain of PKR are shown as ribbon diagrams (on the right) with the side chains of the E33 and E42 as phosphor-mimetic mutations of S33 and T42, and putative associating K261 and R262 residues in the α0-helix on the kinase domain indicated. The orientation of the protein-docking interface shown as a ribbon diagram (on right) replicates that shown in the space-filling diagram of the complex (on the left).

As the structures of the PKR kinase domain and the RNA-binding domain are known, we were in a position to computationally model the putative intra-molecular interaction. We used the 3D-Dock suite Version 3.0, as it allows docking solutions to be filtered based on the prior knowledge of residues known to be involved in the interaction^30^,^31^. The program scans for all possible positions of the two molecules, sorts solutions based on surface complementarity and electrostatic potential, then ranks the docking solutions based on the empirical pair potential matrix derived from 90 non-homologous interfaces found in Protein Data Bank^32^. The S33 and T42 residues were altered to glutamic acids to mimic the phosphorylated forms of these residues and the structure of the RNA-binding domain (RCSB file PDB: 1QU6) was randomly rotated and docked to a hypothetical monomeric kinase domain, which was derived from the structure of the active dimeric kinase domains in complex with eIF2α (RCSB files PDB: 2A1A). The top-ranked solution in which the residues E33 and E42 are involved in the interaction was 22^nd^ following RPScore (Fig. 5). In this solution, the 33 and 42 residues in RBM1 are in proximity to a lysine at position 261 in the α0-helix within the N-lobe of the kinase domain that mediates dimerization, although there is no engagement of residues that have been demonstrated to directly participate in dimerization of PKR protomers. However, K261 immediately precedes an arginine residue that is crucial to configuring the PKR dimer (Fig. 5)^22^. In the PKR dimer, the R262 residue interacts with an asparagine residue number 266 in the second protomer of the complex. As this interaction does not occur in the monomeric protein, the orientation of this R262 residue within the α0-helix may differ from the structure we have used to model the association with RBM1. Accordingly, we tested the significance of residues at both position 261 and 262 to the intra-molecular association in PKR in the following experiments.

### Visualizing molecular interaction of PKR

The association between the RNA-binding and kinase domains was visualized using a fluorescence bimolecular complementation assay. Constructs were differently tagged with the split N- or C-terminal portions of the Venus fluorophore (coded V1 or V2), so that an association between the separately tagged peptides restores the full-length fluorophore to produce a fluorescent signal. These assays were conducted in HEK293 cells in which the endogenous PKR was reduced by RNA interferences as described by us previously^33^.

**Figure 6:**
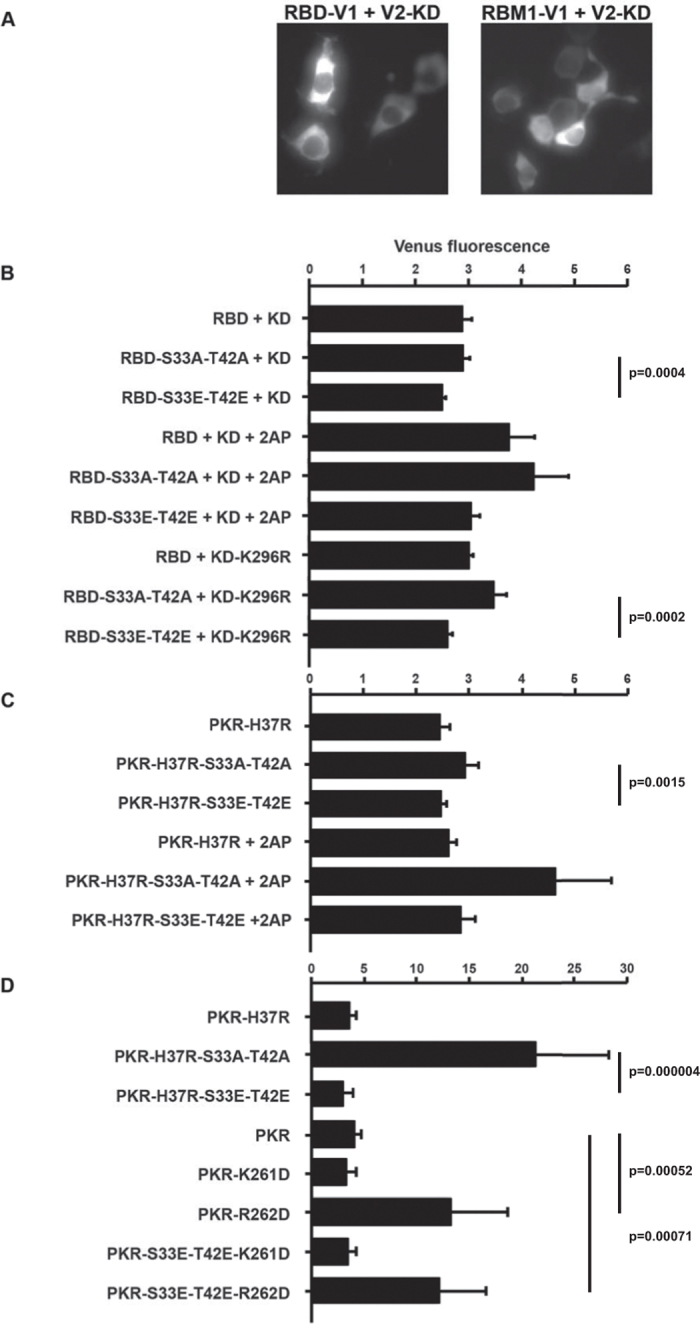
Phosphor-S33 and -T42 residues control the association between RBM1 and the kinase domain and PKR dimerization. (**A**) Micrographs showing Venus fluorescence produced by peptide association in HEK293 cells expressing the RNA-binding domain (RBD) or just RBM1 and the kinase domain (KD) with split Venus as C-terminal V1 and N-terminal V2 tags, respectively (using 20 ng/well of each plasmid construct). (**B**) A quantitation of Venus fluorescence produced by an association between the indicated RNA-binding domain (RBD) constructs and the truncated kinase domain (KD) of PKR tagged with separate halves of the split Venus fluorophore as C-terminal V1 or N-terminal V2 tags, respectively (using 20 ng/well of each plasmid construct). The effect of kinase activity was examined by treatment with 5 μM of the inhibitor 2-aminopurine (2AP) and by using the phosphor-transfer mutant kinase domain (KD-K296R) (n = 7 for the cells expressing the wild-type KD, n = 6 for 2AP-treated cells and n = 5 for experiments with the KD-K296R construct). (**C**) A quantitation of Venus fluorescence produced by dimerization of the indicated full-length PKR constructs and the truncated kinase domain tagged with separate halves of the split Venus fluorophore as N-terminal V1 or V2 tags, respectively (using 20 ng/well of each construct). The histidine residue number 37 was mutated to an arginine (H37R) to reduce RNA-binding to RBM1. The effect of kinase activity was examined by treating transfected cells with 5 μM of 2AP (n = 7 for the cells untreated and n = 3 for cells treated with 2AP). (**D**) A quantitation of Venus fluorescence produced by dimerization of the indicated full-length PKR constructs and the truncated phosphor-transfer mutant kinase domain (KD-K296R) with N-terminal V1 or V2 tags, respectively (using 20 ng/well of full-length PKR and 30 ng/well of the truncated KD). The lysine and arginine resides number 261 and 262, respectively, within the α0-helix of the kinase domain, are mutated to aspartic acids (K261D and R262D) to test the involvement of either residue in the interaction with the 33 and 42 residues in RBM1 (n = 7). Error bars show the SEM in all graphs. Student’s *t* test was used to calculate the *p* values.

Venus fluorescence was produced in cells expressing the RNA-binding domain separately tagged with the split Venus fluorophore. Accordingly, we initially assessed the association between the RNA-binding domain and a truncated kinase domain to circumvent this signal from either homo-oligomerization of RBMs or, co-localization of the domain on RNA. A fluorescent signal was generated by co-expression of the RNA-binding domain and the kinase domain tagged with the split Venus tags (Fig. 6A). This signal was sustained with a construct expressing only RBM1, thereby demonstrating that the signal generated with the entire RNA-binding domain was not merely due to RBM2 (Fig. 6A). Mutating the phosphor-residues to glutamic acids (RBD-S33E-T42E) reduced the peptide association compared to the wild type and alanine mutant construct (RBD-S33A-T42A), as measured by the relative levels of Venus fluorescence (Fig. 6B). This difference became more conspicuous when kinase activity was inhibited by treatment with the inhibitor 2-aminopurine or by mutating the phosphor-transfer residue (K296R) in the kinase domain (Fig. 6B).

Our preceding data predict that the RNA-binding domain primarily associates with the kinase domain as a substrate, then after phosphorylation, in an alternative inhibitory conformation. To try to distinguish these alternatives, we examined fluorescence generated by dimerization of the full-length PKR with the truncated kinase domain. As before, we examined the consequence of kinase activity using the inhibitor 2-aminopurine. Additionally, we sought to reinforce the putative intra-molecular interaction by reducing RNA-binding to RBM1. Directed by structural and mutagenic analysis of RBMs, we mutated the histidine residue number 37 (H37K) within the GPxH motif from the β1-β2 loop of RBM1 (Fig. 1B)^34^,^35^,^36^. Venus fluorescence produced from the association between the full-length and the kinase domain of PKR tagged with the split Venus show that mutating the phosphor-residues affects protein dimerization and this was more conspicuous when kinase activity was inhibited (Fig. 6C). Consistent with the proposition that charging of the S33 and T42 residues by phosphorylation enhanced an intra-molecular interaction with the dimer interface on the kinase domain, the phosphor-mimetic mutant (PKR-S33E-T42E) showed significantly lower fluorescence generated by dimerization than the non-phosphorylatable mutant (S33A-T42A) (Fig. 6C). This indicates that the phosphor-residues in RBM1 repress formation of the kinase dimer. This effect was more manifest when dimerization was tested against the phosphor-transfer mutant (K296R) kinase domain (Fig. 6D). Moreover, mutating the R262 (R262D), but not K261, residue similarly increased dimerization as measured by Venus fluorescence (Fig. 6D). Furthermore, this change (R262D) disrupted the check to dimerization instigated by introducing glutamic acid residues at position 33 and 42 within the RBM1 (Fig. 6D). These data support an association involving the R262 residue in the kinase domain and the phosphor-residues S33 and T42 within the RBM1.

### S33 and T42 phosphor-residues control PKR activity

**Figure 7:**
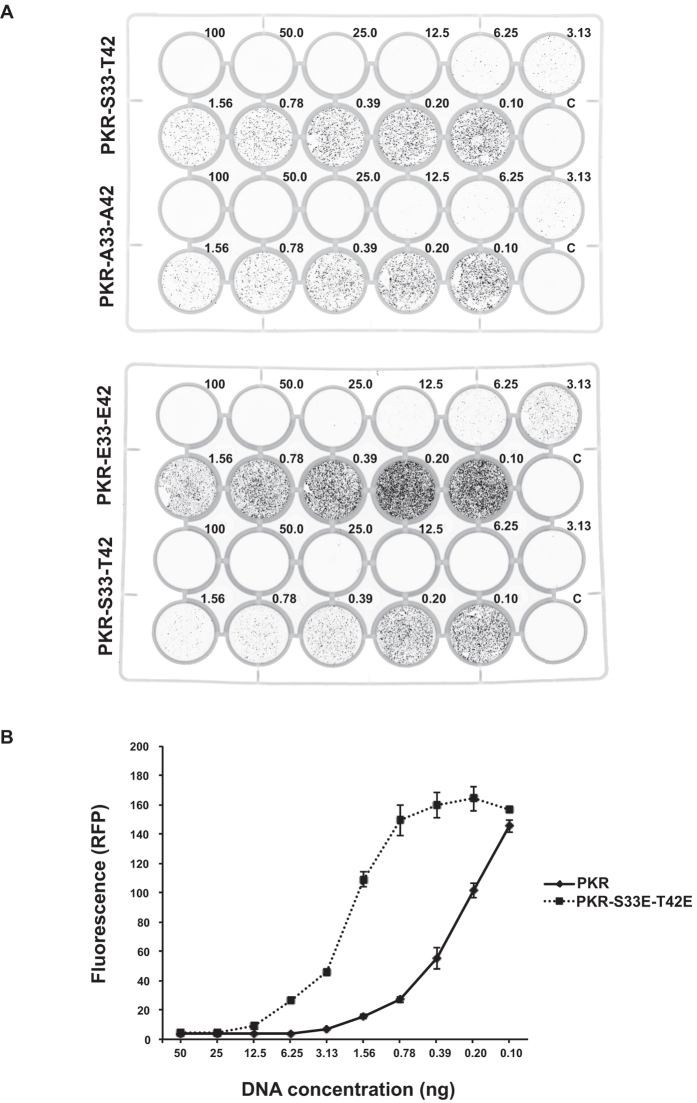
S33 and T42 phosphor-residues control PKR activity. (**A**) Representative images of cell culture plates demonstrating RFP expression in HEK293 cells transfected with an RFP-reporter of protein translation (20 ng/well) and the designated amounts of the indicated PKR constructs (ng/well). The relative effect of mutating S33 and T42 to alanine (A33-A42) is compared to the wild type (S33-T42) in the upper plate and the glutamic acid phosphor-residue mutant (E33-E42) is compared to wild-type PKR in the lower plate. (**B**) A quantitation of the activity of the wild-type (S33-T42) and glutamic acid phosphor-residue mutant (E33-E42) PKR constructs transfected into HEK293 cells, as assessed by their relative control of the RFP reporter of protein translation. The graph shows the total fluorescence per well produced in replicated experiments as shown in (**A**). The data are produced with PKR constructs generated for the bimolecular complementation assays and are expressed with an N-terminal V1 tag. The response of constructs alternatively tagged with the V2 split Venus are shown as Supplementary Figure S5. Error bars show the SEM. Student’s *t* test was used to calculate the *p* values. For all data points, except those for plasmid DNA concentrations of 50, 25, 0.2 and 0.1 ng/well, *p* values were <0.001 (n = 4).

Dimerization is an essential step in forming the active enzyme and so the role of the phosphor-residues in RBM1 in regulating this state would be anticipated to affect kinase activity. To demonstrate the consequence of this, we measured the effect on protein translation using a fluorescent reporter that we have described previously^33^. Mutating the residues at the 33 and 42 positions to glutamic acids, to impose an intra-molecular interaction, inhibits PKR-dependent control of the initiation of protein translation as measured by this reporter (Fig. 7 and Supplementary Figure S5). These findings demonstrate that the S33 and T42 phosphor-residues regulate the activity of PKR.

## Discussion

Collectively the data identify that the S33 and T42 are phosphorylation sites that affect the state of the enzyme, as demonstrated by altered binding of ATP-homologue and peptide ligands at the kinase domain, changed proteolysis of the linker region, and by masking of RNA-binding at the amino-terminus. As the masking of RNA-binding in the monomeric molecule was relieved by protomer dimerization, it suggested an intra-molecular interaction between RBM1 and the established dimer interface at the N-lobe of the kinase domain. Mutagenic analysis confirms modelling that predicted the association between specific residues within RBM1 and the N-lobe of the kinase domain. Correspondingly, we demonstrate that the phosphor-residues inhibit dimerization and repress PKR-dependent control of protein translation.

We are not aware of another instance whereby auto-phosphorylation has been shown to repress kinase activity, although inhibition by a second kinase has been identified. For instance, the proto-oncogene tyrosine-protein (Src) kinases are phosphorylated at their carboxyl-terminus by the c-Src tyrosine protein kinase. These phosphorylated residues then associate with the protein’s first Src homology (SH) domain, which stabilizes an association between the second SH domain and residues that link these to the kinase domain. This then induces an association between tryptophan residue number 260 in the linker and αC-helix in the N-lobe of the kinase domain, which represses kinase activity^37^. We suggest that the auto-inhibition of PKR has parallels with this mechanism. The linker of PKR does not fulfil the same role as it does for the Src kinases, as the function of the W260 residue in the Src kinases appears to be delivered by an isoleucine residue (number 288) within the N-lobe of the PKR kinase domain^22^,^37^. However, there is evidence that the PKR linker participates in auto-regulation. This would seem to constitute more than merely tethering the RNA-binding domain, as modifying residues in the linker alters kinase activity, as reported by others^24^,^38^. Residues in the linker may modulate the associations of the RBMs with the kinase domain, as has been demonstrated for residues adjoining RBMs from other proteins^39^. Alternatively, residues within the linker may directly affect activity by associating with the catalytic pocket of PKR, as is suggested by their phosphorylation, as detected here and as reported by others^15^.

In parallel with the SH domains of Src kinases, we propose that PKR is auto-inhibited through an intra-molecular interaction that engages both of its N-terminal RBMs. For the RBMs to co-operatively repress kinase activity, these homologous peptides must interact with separate surfaces on the kinase domain. Residues that vary between RBM1 and RBM2, between positions 119 and 129 of RBM2, likely determine this specificity. Consistent with these residues mediating discriminatory interactions, this variance is conserved in RBM2 but not RBM1 in PKR from separate species, or RBMs from other proteins (Supplementary Figure S1). As some of these residues directly associate with RNA^34^,^36^, this variance implies that there is an opposing functional imperative for this peptide^40^.

Analysis by others had demonstrated that the RBM2 associates with the C-lobe of the kinase domain in a charged region between the αD, αE and αG-helices^6^,^8^, which coincides with the eIF2α docking site^22^. Our analysis predicts that the RBM1 interacts with the N-lobe of the kinase domain. Exchange spectroscopy by Gelev *et al*.^8^ detected that the RBM1 induced chemical shifts in residues in the N-lobe of the kinase domain (K291-K296). This experimental evidence was somewhat tenuous, possibly because S33 and T42 were unphosphorylated in the kinase-dead protein that was used. As an interaction was detected in the absence of the phosphorylation of S33 and T42, it indicates that additional residues participate in the interaction.

It had initially been contended that only RBM2 instigated auto-inhibition and that RBM1 must remain free in order to initiate RNA binding that then recruits RBM2 to relieve auto-inhibition. However, we propose that the association between RBM1 and the kinase domain does not involve the same face of the molecule that binds RNA, as is the case for RBM2. Instead we contend that the intra-molecular interaction is regulated by phosphor-residues within the domain’s β1–β2 loop. A structure of the RBM from the mitochondrial ribosomal protein L44, in association with the 39S ribosomal subunit, supports a role for residues in this loop in interactions^41^. This alternative orientation would retain the ability of RBM1 to bind RNA when associated with the kinase domain, thereby enabling relief of auto-inhibition.

Our demonstration that phosphor-residues affect intra-molecular interaction offers an explanation for why earlier studies identified only a role for RBM2 in auto-inhibition and, also, how more recent findings controverted regulation of PKR by auto-inhibition. The initial studies used peptides that were separately expressed and purified and/or used the kinase-dead construct (K296R), and so did not account for phosphorylation. More recent studies that refute auto-inhibitory control of PKR used a wild-type construct that is co-expressed with a lambda protein phosphatase (PKR-PPase)^42^. In this system, PKR undergoes auto-phosphorylation that is then substantially removed. Accordingly, phosphorylation of the S33 and T42 residues is unlikely to be retained. Also, the removal of phosphates in this system is incomplete (Supplementary Figure S6). These observations are consistent with auto-phosphorylation driving conformational change of the kinase domain, making phosphor-residues inaccessible to the phosphatase^17^. This suggests that the PKR-PPase molecule is in a partially active confirmation, and so may not reliably replicate the activation mechanisms of the kinase.

There is precedence for inhibition of PKR activity through protein association at the N-lobe, as we propose for RBM1. The chaperone protein DNAJC3 (also called P581PK) associates with a similar region of the kinase domain (within the residues 244–296), as predicted for RBM1^43^. DNAJC3 reportedly blocks PKR dimerization^44^. A precedent for inhibition of PKR activity at the RBM2 docking position is embodied by the vaccinia virus K3 protein (the product of the viral K3L gene), which associates with an overlapping face of the C-lobe. However, K3L does not alter the auto-phosphorylation of PKR and is only effective in diminishing substrate phosphorylation^43^,^45^,^46^, consistent with this face of the kinase domain associating with eIF2α^22^. This may speak to the necessity for co-operative activity of the RBMs to maintain an inactive kinase conformation. Meharena *et al*.^1^ identified four separate inactivating conformations of the kinase domain that they termed: ‘DFG-out’ and ‘HRD-out’, pertaining to the attitude of these sequence motifs within the activation segment and activation loop, respectively; ‘αC-helix-out’, which pertains to misalignment of residues that connect the separate lobes of the kinase domain; and ‘twisted lobe’, which pertains to the spatial alignment of the N- and C-lobes of the kinase domain. High-resolution structures of the related eIF2α kinase, the general control nonderepressible 2 (GCN2), demonstrate repositioning of the αC-helix and biochemical experiments support that regulation of this element controls activity^47^,^48^,^49^. The ‘αC-helix-out’ conformation is likely to also be representative of the inactive PKR molecule. Notably, mutations that disrupt PKR dimerization inactivate the molecule^50^. This highlights the significance of RBM1 docking at the kinase dimerization interface. The association of RBM2 on the alternative face, at the C-lobe of the kinase domain, could also repress dimerization by impeding the formation of transitional states of PKR. Although PKR protomers associate in a back-to-back orientation in the active enzyme^22^, Li *et al*.^51^ identified that introducing a phosphor-mimetic mutated at the essential catalytic residue (T446D) in the kinase-dead molecule enabled them to isolate a dimeric molecule that adopted an alternative face-to-face orientation.

Alternatively, it has been proposed that RBM2 obstructs an undetermined allosteric effect in the activation of PKR. The proposed positioning of RBM2 and the rigidity of much of the C-lobe in this region would seem to prescribe that such a mechanism involves residues within the flexible catalytic loop of the kinase domain. Accordingly, RBM2 might prevent essential auto-phosphorylation of the catalytic site (T446), thereby controlling allosteric effects. Perturbing an interaction between the catalytic loop and residues in the αC-helix could also prevent reposition of the αC-helix to establish the active kinase confirmation. Notably, replacing the activation loop of PKR with that from the constitutively active phosphorylase kinase 1, which contains a charged glutamic acid (E182) at the catalytic site, rescues the activity of dimerization mutants^9^. This is likely due to stabilization of the interaction between the catalytic loop and residues in the αC-helix that are evident in the active PKR structure^50^.

In summary, we propose a model for the regulation of PKR in which auto-phosphorylation of the S33 and/or T42 residues in RBM1 instigates an association with the N-lobe of the kinase domain that stabilizes a second association between the RBM2 and the C-lobe of the kinase domain. This co-operative, intra-molecular association restrains conformational changes required to constitute the active kinase molecule. The mechanism implies that PKR is inherently active and so must be constrained. We believe this is in keeping with the sensor and effector function of PKR within the innate immune response. It is also in keeping with the identification of a number of cellular suppressors that appear to target the monomeric PKR protein. The proposed mechanism would appear to be common to the other eIF2α kinases, with the Heme-regulated eIF2α kinase restrained by heme-binding, the PKR-like endoplasmic reticulum kinase by binding of the Heat Shock 70 kDa protein 5 (also called BIP and GRP78) and GCN2 by an intra-molecular auto-inhibition, although the precise mechanisms of suppression for these proteins is still unclear. As the cytosolic inhibitor of PKR, DNAJC3, has also been reported to suppress the activity of two other eIF2α kinases^52^,^53^, it suggests a conserved regulatory process. By identifying mechanisms that regulate PKR, these findings indicate a strategy to control kinase activity. More broadly, resolving auto-inhibitory mechanisms of other kinases constitutes an informed strategy to develop discriminatory inhibitors that are based on a functioning process that is specific for each kinase.

## Methods

### Reagents

2-Aminopurine was purchased from GE Healthcare Life Sciences. Anti-PKR (sc-6282) and -eIF2α (sc-81261) antibodies were from Santa Cruz Biotechnology, Inc. (USA). The anti-pS^51^-eIF2α (447289) was from Invitrogen. *Escherichia coli* BL21 was from Sigma-Aldrich (USA). Caspase-3 was used as recommended by the manufacturer (Immunochemistry Technologies). Cells were transfected with Lipofectamine^®^2000 (ThermoFisher Scientific). Specific residues within PKR were altered by polymerase chain reaction (PCR), using mutagenic oligomers and the PfuTurbo DNA Polymerase (Agilent Technologies). PKR constructs were generated by point mutations introduced by PCR.

### Immunoanalysis

Western blot analysis was conducted by transferring whole-cell lysates to Immobilon-FL, probing with primary antibodies followed by Alexa 680 nm or IRdye800 nm secondary antibodies, then visualizing by Licor-Odyssey (Biosciences). Primary antibodies consisted of the rabbit polyclonal eIF2α[pS52]PAb, eIF2αPAb (Invitrogen), rabbit monoclonal antibody YE350 (Abcam), rabbit polyclonal EIF2B2 (Abcam) and mouse monoclonal GAPDH (Abcam). Western blots were quantified with NIH Image J using sum intensity of the OD profile plots, with background correction calculated per lane.

### Mass spectrometry

Purified recombinant PKR proteins were trypsin digested, and analysed using matrix-assisted laser desorption ionization (MALDI) time of flight (TOF) mass spectrometry and analytical high performance liquid chromatography (HPLC) coupled with electrospray mass spectrometry (ESI-MS). Potential phosphor-peptides were identified by tandem mass spectrometry by searching for a characteristic mass loss equating to the loss of the phosphate group. To detect low intensity phosphor-peptides, the digest was then applied to a commercial phosphor-peptide affinity enrichment kit, and enriched phosphor-peptides were subsequently eluted and analysed by the above mass spectrometry methods. To characterize the site of phosphorylation, the phosphor-peptides were analysed on an ESI mass spectrometer with electron transfer dissociation (ETD) capability, giving sequence-specific information with the phosphor-peptides remaining intact. The phosphor-analysis was conducted on wild type and, as a control for auto-phosphorylation activity, kinase-dead (K296R) molecules.

### Bimolecular complementation assays

Bimolecular complementation assays were performed as previously described in HEK293:shPKR cultured in DMEM supplemented with 10% fetal bovine serum under puromycin (10 μg/ml) selection, at 37 °C in a 5% CO_2_ humidified incubator^54^. Cells (0.5 × 10^5^/well) were plated into a 24-well plate (BD Falcon), then transfected with separately tagged PKR constructs the next day and Venus fluorescence detected after 48 h. Total fluorescence was quantified using ImageJ^55^.

### Assays of protein translation

A fluorescent reporter was generated that expressed monomeric red fluorescent protein (RFP) under control of an RNA polymerase III U6 promoter enhanced with the RNA polymerase II cytomegalovirus promoter in a pBluescript (Stratagene) backbone plasmid. Control of translation by PKR was assessed by co-transfecting HEK293 cells with this reporter and plasmid that expressed different PKR constructs, then RFP production was measured after 60 h using a Typhoon Trio (GE Healthcare).

### Preparation of recombinant proteins

His-tagged PKR proteins and the tobacco etch virus TEV protease were prepared in BL21-basic *Escherichia coli* cells as described previously^14^.

### Fluorescent measures of ligand binding

Purified single (K296R) or triple (K296R-S33E-T42E) mutant PKR proteins were incubated at molar ratios of 0.2, 0.5, 1.0, 2.0, 5.0, 10, 20, 50, 100 and 200 with 100 μM of 2-aminopurine nitrate salt (SIGMA) or, alternatively, FITC-P2-peptide in 100 μl of buffer (10 mM Hepes pH 7.0, 50 mM NaCl, 1 mM EDTA, 1 mM dithiothreitol (DTT)), at room temperature for 20 min. Fluorescence was measured in an opaque, black 96-well plate (Millipore), at a 5 nm spectral band pass with excitation and emissions set to 310 and 340–550 nm, respectively, for 2-aminopurine, or 413 and 480–580 nm for the FITC-peptide, using a SpectraMAxi3 (Molecular Devices) microplate reader.

### RNA-binding assay

Purified preparations of single or triple mutant PKR proteins were incubated at molar ratios of 0.5, 1.0, 2.0, 5.0 and 10 with 50 ng of a duplex 57 nucleotide RNA species derived from the human immunodeficiency virus type I (HIV-1) trans-activation response element (TAR). Protein-RNA complexes were formed in 20 μl of a binding buffer (0.5 × TBE, 0.5% NP40, 0.5% Brij35, 0.5 ng/μl heparin, 0.5 mM argininamide, 10% glycerol) for 1 h at 4 °C, centrifuged at 10,000 *g* for 1 min, then electrophoretically separated through a native 10% polyacrylamide gel at 200 V, conducted at 4 °C for 20 min in TBE buffer. RNA was stained with a 10,000× dilution of SYBR Gold (ThermoFisher Scientific) in 1 × TBE for 10 min, then visualized with a Typhoon image scanner (GE Healthcare Life Sciences) using excitation with the 488 nM blue laser and emission with the LPB filter (≥510 nM). Images were analysed using ImageQuant 1.2 software (Molecular Dynamics).

### Proteolysis

Human recombinant caspase-3 (Abcam) was diluted to 1 U/μl aliquots in 1× phosphate-buffered saline (PBS) with 15% glycerol and stored at −70 °C before use. His-tagged PKR peptides were purified and 15 μg of protein was digested with caspase-3 in digestion buffer (50 mM Hepes pH 7.2, 50 mM NaCl, 0.1% Chaps, 10 mM EDTA, 10 mM DTT, 5% glycerol) or, alternatively, TEV in digestion buffer (50 mM Tris-HCl pH 8, 0.5 mM EDTA and 1 mM DTT) at 37 °C with caspase-3 (1 U/μl), or TEV (1 U/μl), respectively, for 80 min. His-tagged peptides were recovered by incubation with 1 μl Ni-Sepharose beads (GE Healthcare) at 4 °C overnight with a gentle rotation and centrifugation at 500 rpm for 1 min in a microcentrifuge. The pellet was resuspended in 100 μl loading buffer and 30 μl was loaded onto a polyacrylamide gel for electrophoretic separation and peptides detected by immunoblotting.

### Isoelectric focussing

Two-dimensional gel electrophoresis used the ZOOM Benchtop Proteomic System (ThermoFisher Scientific).

### Circular dichroism spectral analysis

Circular dichroism spectra were measured at 20 °C using a Jasco J815 CD spectrophotometer. All scans were run on proteins concentrated to 400 μg/ml in PBS. Scans were run between 190 and 260 nm and the average ellipticity of triplicate scans recorded. Data were converted to mean residue ellipticity (MRE) by the equation of Correa and Ramos^56^. MRE were plotted and compared using GraphPad Prism.

### Statistical analysis

The statistical applications within Microsoft Excel or the program R-2.14.0 (http://R-project.org) were used to analyse the data. All statistical analysis was performed with GraphPad Prism or Microsoft Excel software. Differences were calculated using the unpaired, two-tailed *t*-test for pairwise comparisons, or by linear regression calculated for each genotype, for the temporal data.

## Additional Information

**How to cite this article**: Wang, D. *et al*. Auto-phosphorylation Represses Protein Kinase R Activity. *Sci. Rep.* **7**, 44340; doi: 10.1038/srep44340 (2017).

**Publisher's note:** Springer Nature remains neutral with regard to jurisdictional claims in published maps and institutional affiliations.

## Supplementary Material

Supplementary Figures S1-S6
