# Supplementary material for: Auto-phosphorylation Represses Protein Kinase R Activity
Source: Sci Rep. 2017 Mar 10;7:44340. doi: 10.1038/srep44340 (PMC5345052; doi:10.1038/srep44340)
Supplement: Supplementary Figures S1-S6 [file srep44340-s1.pdf]

Auto-phosphorylation Represses Protein Kinase R Activity

Die Wang[1], Nicole A de Weerd[2,4], Belinda Willard[3], Galina Polekhina[1], Bryan RG Williams[1,4] and Anthony J Sadler[1,4,*]

[1]Centre for Cancer Research, Hudson Institute of Medical Research, Clayton, Victoria 3168, Australia.

[2]Centre for Innate Immunity and Infectious Diseases, Hudson Institute of Medical Research, Clayton, Victoria 3168, Australia.

[3]Proteomics and Metabolomics Laboratory, Lerner Research Institute, Cleveland Clinic, Cleveland, Ohio 44195, USA.

[4]Department of Molecular and Translational Science, Monash University, Clayton, Victoria 3168, Australia.

[*]Corresponding author: anthony.sadler@hudson.org.au

Supplementary Figure S1. Conservation of the phosphor-S33 and -T42 residues in RBMs.

A more extensive alignment of the amino acid sequence of RBMs than that shown in Figure 1B, which includes peptide sequences from *Homo sapiens* (Hs) proteins other than PKR as indicated; RHA=ATP-dependent RNA helicase A, TRBP1=TAR (HIV-1) RNA-binding protein 1, STRBP=spermatid perinuclear RNA binding protein, ADAR1=double-stranded RNA-specific adenosine deaminase-1, PACT=PKR protein activator, ILF3=interleukin enhancer binding factor-3.

```
                                      33         42             52      60
                                       |          |              |       |
HsPKR-RBM1      FMEELNTYRQKQG-VVLKYQELPNSGPPHDRR---FTFQVII-----D--GREF-PEGEGRSKKEAKNAAAKLAVEILN
PtPKR-RBM1      FMEELNTYRQKQG-VVLKYQELPNSGPPHDRR---FTFRVII-----D--GREF-PEGEGRSKKEAKNAAAKLAVEILN
MnPKR-RBM1      YMEELNKYRQKQG-VSLSYQELPNTGPPHDRR---FTFQVVT-----D--RRTF-PEAEGRSKKEAKNAAAKLAVDILN
SsPKR-RBM1      YIEELNKYQQKND-VILKYRELCKTGPAHNLR---FTYQVVI-----D--EKEF-PKAEGRSKKEAKNAAVKLAFEIIN
MmPKR-RBM1      YMDKLNKYRQMHG-VAITYKELSTSGPPHDRR---FTFQVLI-----D--EKEF-PEAKGRSKQEARNAAAKLAVDILD
BtPKR-RBM1      YIEELNKYQQKNG-VEVRYCELAKTGPPHNFR---FTYQVII-----D--DKEY-PKAEGRSKKEAKNAAAKLALEIIN
MaPKR-RBM1      YMDKLNKYHQKHR-VTITYKHLFTTGPPHDRR---FTFQVII-----D--GEEF-PEAEGKTKQEAKNAAAKLAVDTLN
EcPKR-RBM1      FIEELNKYRQKHN-VVLDFRELSKTGPPHDLT---FTFRVII-----N--GREF-PEAEGKSKKEAKNAAAKMAVEILR
PaPKR-RBM1      FLEELNKYRQKNN-VEIEYRELSKRGPPHDLR---FTFQVVI-----G--EREF-PEAEGKSKKGAKNAAAKLAVEILN
XtPKR-RBM1      AKGQLITFCIKNG-LAYHFNEVEATGPSHDPR---FTSQVFV-----N--QEKL-GEGQDKKKKGAENIAAKMALLTLK
DrPKR-RBM1      YTSLLNEYQQKTQ-CTVEFEEGPTDGPSHNKR---FTMRAIV-----N--GQKF-PDGTGKTKKEAKQNAAKNALEGLK

HsPKR-RBM2      YIGLINRIAQKKR-LTVNYEQCA-SGVHGPEG---FHYKCKM-----G--QKEY-SIGTGSTKQEAKQLAAKLAYLQIL
PtPKR-RBM2      YIGLINRIAQKKR-LTVNYEQCA-SGVHGPEG---FHYKCKM-----G--QKEY-SIGTSSTKQEAKQLAAKLAYLQIL
MnPKR-RBM2      YIGVVNRIAQKKR-LTVNYEQCT-SGVHGPEG---FHYKCKI-----G--DKEY-GIGTGSSKQEAKQLAAKLALLEIS
SsPKR-RBM2      YIGRINTISQKKN-LSVNYEPCE-PGEDGPEK---FHYKCKI-----G--RKVY-GSGVGSTKQEAKQLAAKQAYEKLQ
MmPKR-RBM2      YIGLVNSFAQKKK-LSVNYEQCE-PNSELPQR---FICKCKI-----G--QTMY-GTGSGVTKQEAKQLAAKEAYQKLL
BtPKR-RBM2      YIGRLNTISQKKN-LCVTYEECK-SKGDGPEG---FHYICKI-----G--QEEY-GSGVGSTKQEAKQLAAKLAYEKIE
MaPKR-RBM2      YIGLVNSYAQKEK-LSVNYQQCA-FNTQSPQR---FCYKCII-----G--LKTY-GIGSGATKQEAKQLAAKEAYQKLP
EcPKR-RBM2      YIGLINTYTQRNQ-LSLNYEQYE-SRDGGPKR---FRCKYKI-----G--WKEY-PIASGSTKQEAKQLAAKLAYDQIS
PaPKR-RBM2      YIGLVNRIAQKAK-LPVNYQLGL--GAGEPGR---FYYTCII-----G--QKKY-NVAVGSTKQKAKQLAAKLAYEQIL
XtPKR-RBM2      YVGKLHELCQKHK-LICKFFD-ECYGLPHIPE---FWCKVVI-----G--EEEF-HKVKGNNKKEAKRKAAYFALMSLK
DrPKR-RBM2      YTCWLNEHSQKSR-LMFKACESTKMDPGNLTRLCTYVCKYVC-----D--DKEF-PEGYGKNKKEAKEAAALRVYEELN

HsRHA-RBM1      VKNFLYAWCGKRK-MTPSYEIR-AVGNKNRQK---FMCEVQV-----E--GYNYTGMGNSTNKKDAQSNAARDFVNYLV
HsRHA-RBM2      AKARLNQYFQKEK-IQGEYKY-TQVGPDHNRS---FIAEMTIYIKQLG--RRIF-AREHGSNKKLAAQSCALSLVRQLY
HsTRBP1-RBM1    PISLLQEYGTRIG-KTPVYDLLKAEGQAHQPN---FTFRVTV-----G--DT-S-CTGQGPSKKAAKHKAAEVALKHLK
HsTRBP1-RBM2    PVGALQELVVQKGWRLPEYTVTQESGPAHRKE---FTMTCRV-----E--RF-I-EIGSGTSKKLAKRNAAAKMLLRVH
HsTRBP1-RBM3    CCRVLSELSEEQA-FHVSYLDIEELSLS--GL---CQCLVEL-----STQPATV-CHGSATTREAARGEAARRALQYLK
HsSTRBP-RBM1    ALMRLNQIR-----PGLQYKLLSQSGPVHAPV---FTMSVDV-----D--GTTY--EASGPSKKTAKLHVAVKVLQAMG
HsSTRBP-RBM2    PVMELNEKR-----RGLKYELISETGGSHDKR---FVMEVEV-----D--GQKF--RGAGPNKKVAKASAALAALEKLF
HsADAR2-RBM1    ALMQLNEIK-----PGLQYTLLSQTGPVHAPL---FVMSVEV-----N--GQVF--EGSGPTKKKAKLHAAEKALRSFV
HsADAR2-RBM2    PVMILNELR-----PGLKYDFLSESGESHAKS---FVMSVVV-----D--GQFF--EGSGRNKKLAKARAAQSALAAIF
HsADAR3-RBM1    ALVQLHELR-----PGLQYRTVSQTGPVHAPV---FAVAVEV-----N--GLTF--EGTGPTKKKAKMRAAELALRSFV
HsADAR3-RBM2    PVVLLNRLR-----AGLRYVCLAEPAERRARS---FVMAVSV-----D--GRTF--EGSGRSKKLARGQAAQAALQELF
HsADAR1-RBM1    PISGLLEYAQFAS-QTCEFNMIEQSGPPHEPR---FKFQVVI-----N--GREF-PPAEAGSKKVAKQDAAMKAMTILL
HsADAR1-RBM2    PVTTLLECMHKLG-NSCEFRLLSKEGPAHEPK---FQYCVAV-----G--AQTF-PSVSAPSKKVAKQMAAEEAMKALH
HsADAR1-RBM3    PVGGLLEYARSHG-FAAEFKLVDQSGPPHEPK---FVYQAKV-----G--GRWF-PAVCAHSKKQGKQEAADAALRVLI
HsPACT-RBM1     PIQVLHEYGMKTK-NIPVYECERSDVQIHVPT---FTFRVTV-----G--DI-T-CTGEGTSKKLAKHRAAEAAINILK
HsPACT-RBM2     PIGSLQELAIHHGWRLPEYTLSQEGGPAHKRE---YTTICRL-----E--SF-M-ETGKGASKKQAKRNAAEKFLAKFS
HsPACT-RBM3     YIQLLSEIAKEQG-FNITYLDIDELSAN--GQ---YQCLAEL-----STSPITV-CHGSGISCGNAQSDAAHNALQYLK
HsILF3-RBM1     ALMRLNQLK-----PGLQYKLVSQTGPVHAPI---FTMSVEV-----D--GNSF--EASGPSKKTAKLHVAVKVLQDMG
HsILF3-RBM2     PVMELNEKR-----RGLKYELISETGGSHDKR---FVMEVEV-----D--GQKF--QGAGSNKKVAKAYAALAALEKLF

BADAVGGOOD
```

Supplementary Figure S2. Phosphor-S33 and -T42 affect the association between PKR and 2AP.

Measures of the comparative binding of 2-aminopurine nitrate (2AP) to the indicated PKR constructs by assessing changed fluorescence from the molecule. The graphs show that the separate PKR constructs differently diminished 2AP-fluorescence at a range of concentrations of PKR.

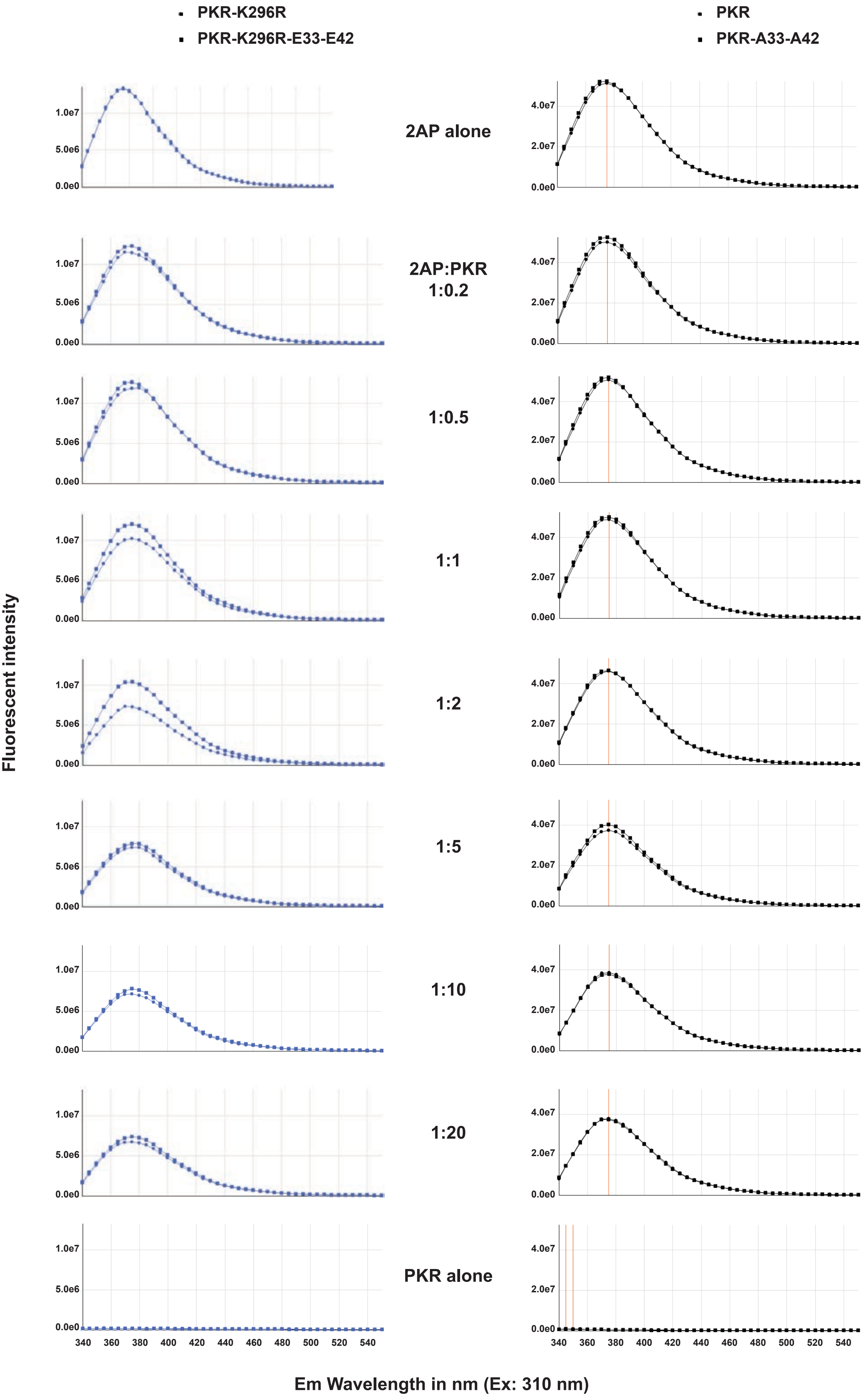

PKR-K296R
PKR-K296R-E33-E42
PKR
PKR-A33-A42
2AP alone
2AP:PKR 1:0.2
1:0.5
1:1
1:2
1:5
1:10
1:20
PKR alone
Fluorescent intensity
Em Wavelength in nm (Ex: 310 nm)

Supplementary Figure S3. SPR is unable to distinguish an association between PKR and sunitinib.

Overlay of sensorgrams from an SPR experiment showing the Response Units (RU) of sunitinib (at 20 μM) flowing across triplicate channels of immobilized kinase-dead PKR (K296R). The vertical dashed line represents the transition between the association phase (0 to 60 sec) and the dissociation phase (61 sec and beyond).

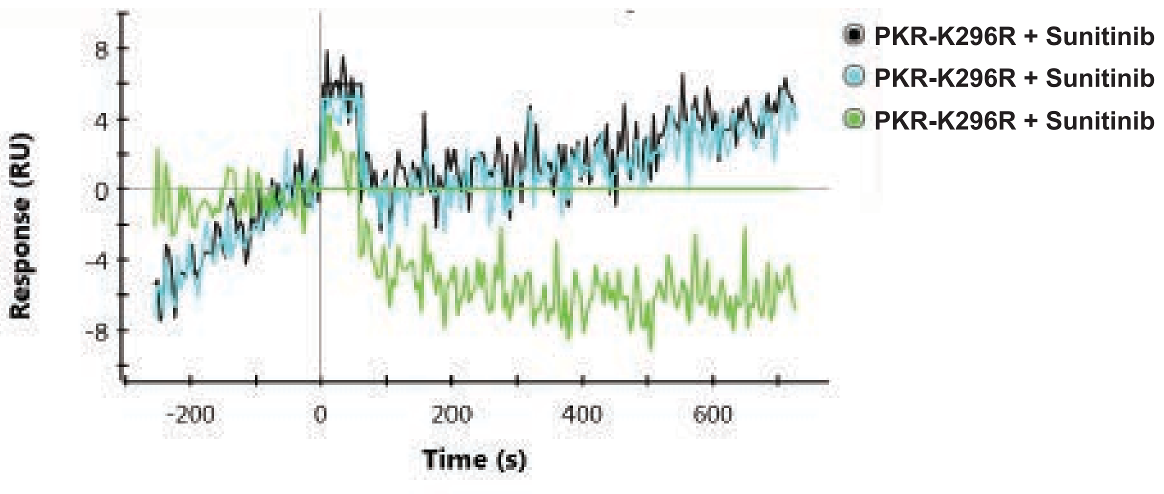

Response (RU)
8
4
0
-4
-8
-200
0
200
400
600
Time (s)
PKR-K296R + Sunitinib
PKR-K296R + Sunitinib
PKR-K296R + Sunitinib

Supplementary Figure S4. Phosphor-S33 and -T42 affect the association between PKR and the P2 peptide.

Measures of the comparative binding of a FITC-tagged peptide (P2) to the indicated PKR constructs by assessing the relative FITC fluorescence. The graphs show that the separate PKR constructs differently affect FITC-P2 fluorescence at a range of concentrations of PKR.

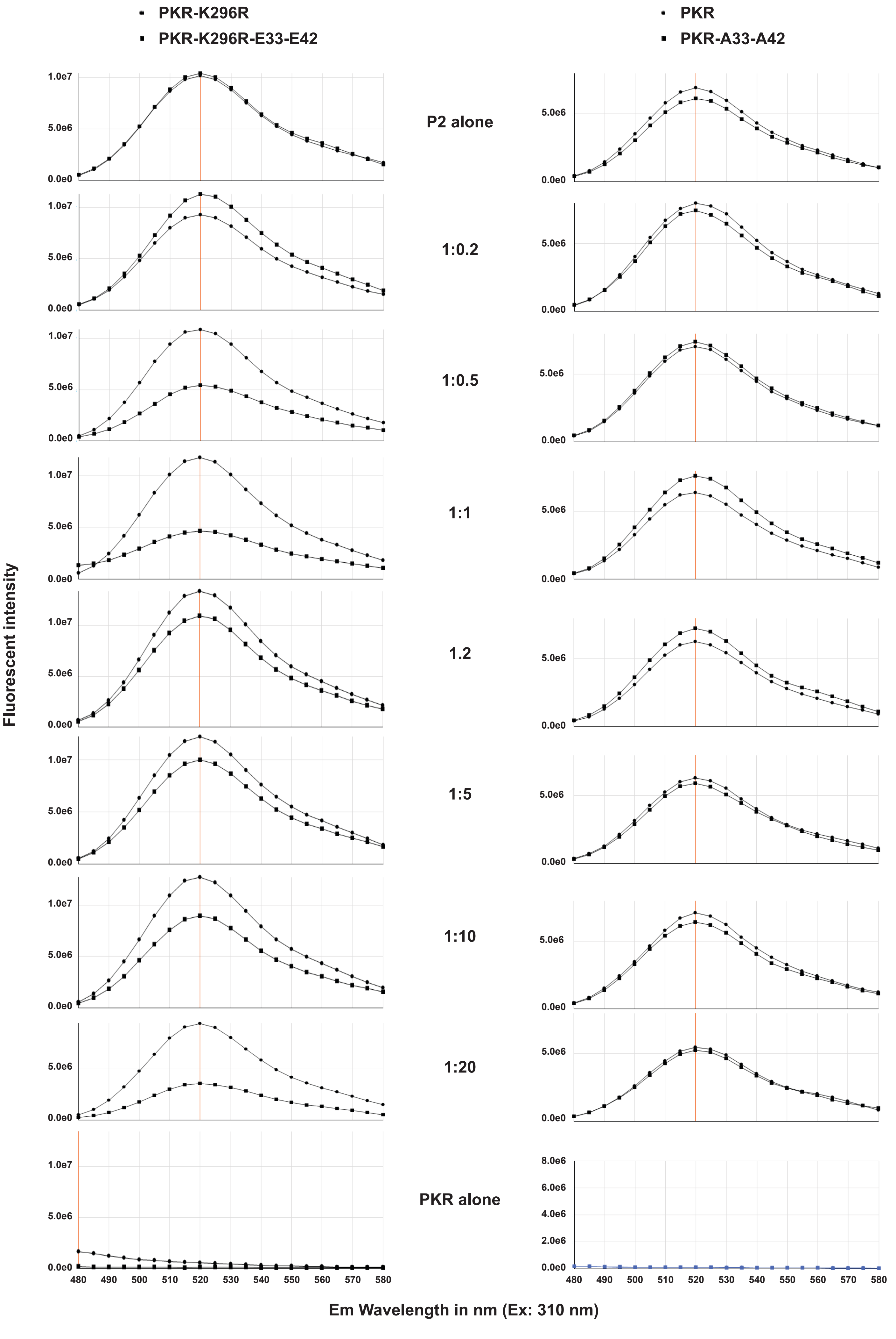

PKR-K296R
PKR-K296R-E33-E42
PKR
PKR-A33-A42
P2 alone
1:0.2
1:0.5
1:1
1.2
1:5
1:10
1:20
PKR alone
Fluorescent intensity
Em Wavelength in nm (Ex: 310 nm)

Supplementary Figure S5. S33 and T42 phosphor-residues control PKR activity. A quantitation of the activity of the indicated PKR constructs transfected into HEK293 cells as assessed by their control of the RFP translational reporter. The PKR constructs are N-terminally tagged with V2 split Venus, in contrast to the V1-tagged PKR constructs used to produce Figure 7B. The separate split Venus tags differently affect PKR activity, so that the V1-tagged PKR is more active than the V2-tagged PKR. The data are also displayed differently to the data shown in Figure 7B, with the x-axis numerically increasing and the y-axis showing fluorescence levels normalized against a kinase-dead PKR construct. The effect of the different PKR constructs for the levels of fluorescence produced have been normalized against that of a kinase-dead (K296R) PKR construct. Student's *t* test was used to calculate the *p* values from these data (n=5, * = $p<0.05$, ** = $p<0.01$).

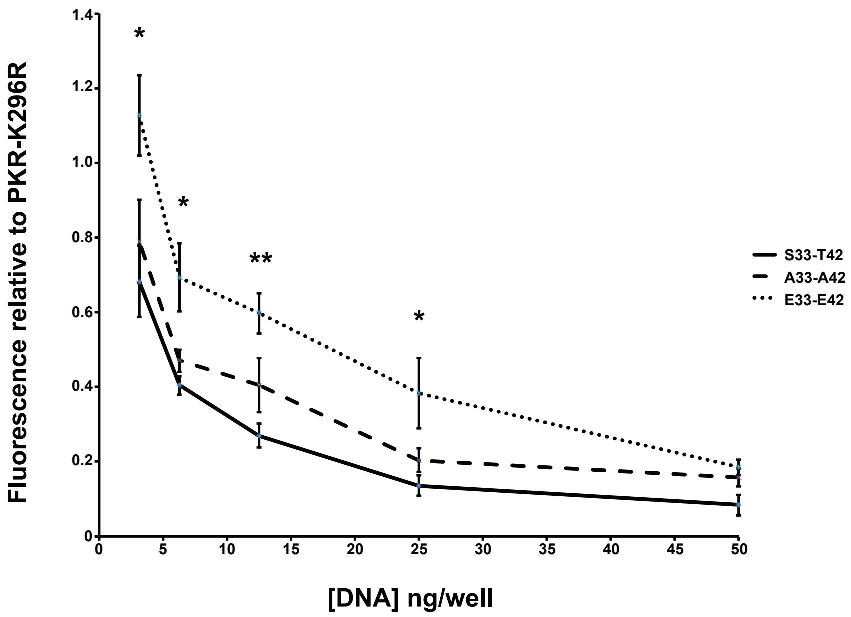

Fluorescence relative to PKR-K296R
[DNA] ng/well
S33-T42
A33-A42
E33-E42

Supplementary Figure S6. The PKR-PPase construct retains auto-phosphorylation.

(**A**) The indicated recombinant PKR proteins electrophoretically separated by their isoelectric point (pI), then visualized with the Coomassie brilliant blue stain. The data show that two independent preparations of the PKR-PPase protein have an intermediate charge between that of the kinase-dead (K296R) and wild-type (WT) proteins, indicating that it retains phosphor-residues, despite the co-expression of the phosphatase.

(**B**) Whole-cell lysates or purified protein preparations (analysed in (**A**)) from *E. coli* that express recombinant human PKR upon induction with IPTG or PKR purified from these lysates were electrophoretically separated then visualized with a phosphor stain (on left) or by Coomassie brilliant blue stain (on right).

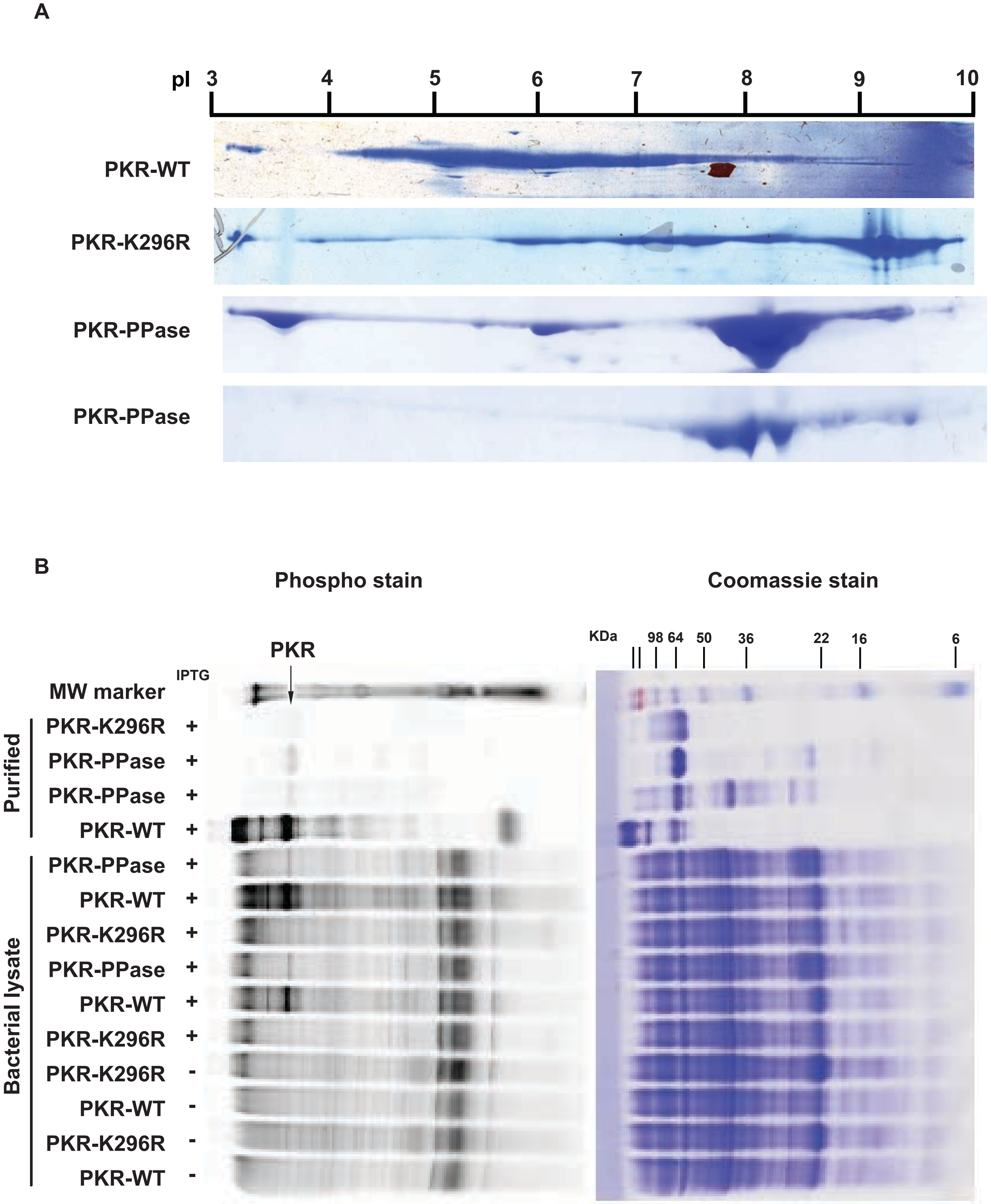
A
pI
3
4
5
6
7
8
9
10
PKR-WT
PKR-K296R
PKR-PPase
PKR-PPase
B
Phospho stain
Coomassie stain
KDa
98
64
50
36
22
16
6
PKR
IPTG
MW marker
Purified
PKR-K296R +
PKR-PPase +
PKR-PPase +
PKR-WT +
Bacterial lysate
PKR-PPase +
PKR-WT +
PKR-K296R +
PKR-PPase +
PKR-WT +
PKR-K296R +
PKR-K296R -
PKR-WT -
PKR-K296R -
PKR-WT -
